# Leprosy reactions: Earlier diagnosis leads to more effective treatment

**DOI:** 10.1590/0037-8682-0375-2019

**Published:** 2020-03-16

**Authors:** Pugazhenthan Thangaraju, Sajitha venkatesan

**Affiliations:** 1 Department of Pharmacology, All India Institute of Medical Sciences (AIIMS), Raipur, Chhatisgarh, India.; 2 Department of Clinical Division, Central Leprosy Teaching And Research Institute, Chengalpattu, Tamilnadu, India.

**FIGURE 1:**
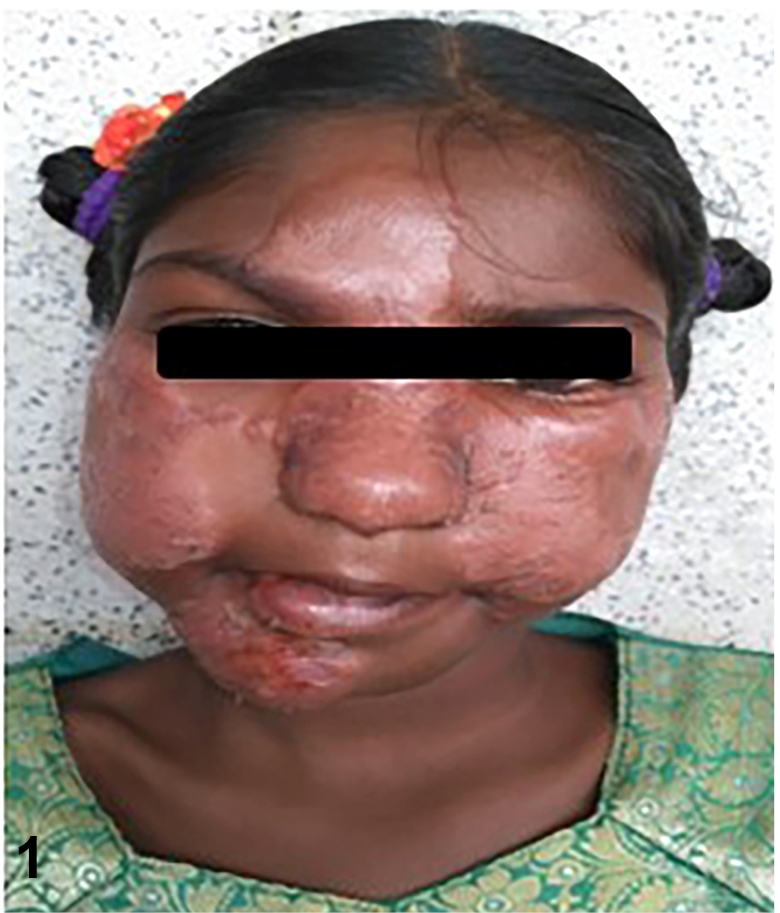
Swelling of the existing lesions on the forehead and cheeks.

**FIGURE 2:**
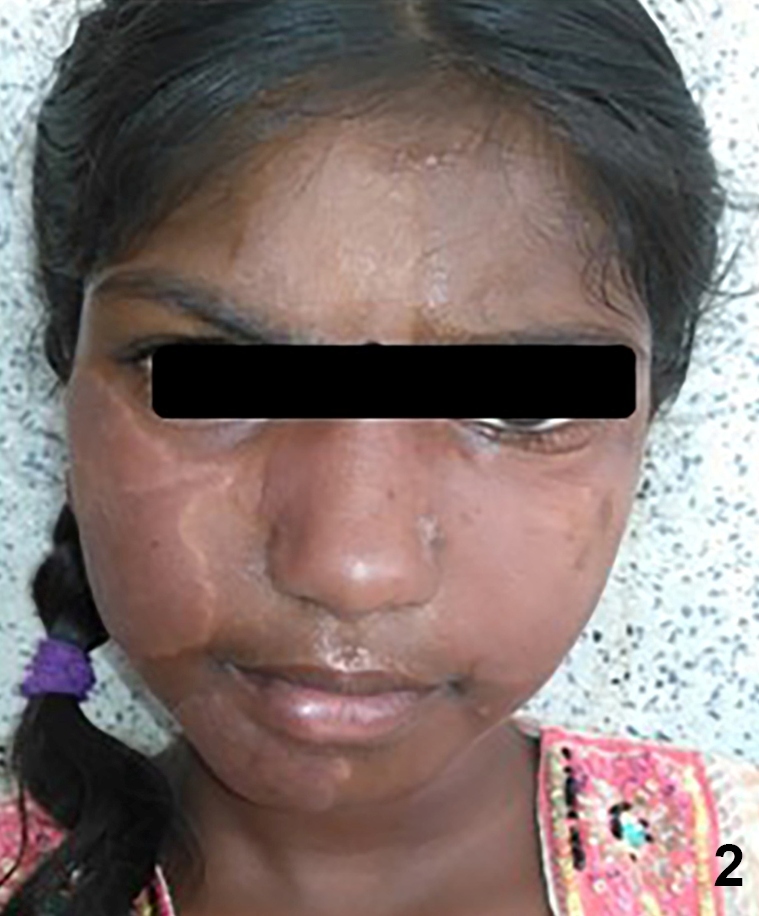
Drastic reduction in the swelling of the lesions.

A 15-year-old child was referred for consultation due toa clinical condition characterized by the swelling of the face, cheek, and ears (Figure 1). The patient was diagnosed with multibacillary leprosy; she had multiple patches on her face and suprascapular region. Three months earlier, a multibacillary multidrug therapy child regimen (MB-MDT C) was initiated at a nearby primary health facility. On examination, the patient had erythematous raised lesions on the forehead, cheeks, and ear lobes. Constitutional symptoms (e.g., fever) were not observed. Blood laboratory investigation showed anemia, leukocytosis, and a high erythrocyte sedimentation rate; other routine investigations were normal. The diagnosis of the type-1 leprosy reaction was based solely on the presentation of previously involved and existing lesions because the patient’s father refused additional investigations (e.g., biopsy). Prednisolone was administered at an anti-inflammatory dose (1 mg/kg of body weight^1^); the patient’s clinical condition improved markedly over a short period. (Figure 2).

The patient’s clinical condition was observed by healthcare providers during a routine visit to her region. As leprosy was diagnostically considered, the case was referred to us. It is strongly recommended that physicians or leprologists responsible for patient care adequately guide basic-level health care teams to identify and quickly refer patients with suggestive leprosy reactions. This will assist in the prompt management and improvement of cases, and prevent the development of disability and mental trauma due to leprosy reactions.
